# Food Insecurity and Nutritional Risk among Canadian Newcomer Children in Saskatchewan

**DOI:** 10.3390/nu11081744

**Published:** 2019-07-29

**Authors:** Ginny Lane, Christine Nisbet, Hassan Vatanparast

**Affiliations:** 1 School of Public Health, University of Saskatchewan, Saskatoon, SK S7N 2Z4, Canada; 2 College of Pharmacy and Nutrition, University of Saskatchewan, Saskatoon, SK S7N 2Z4, Canada

**Keywords:** children, refugee, immigrant, food security, health

## Abstract

Food insecurity continues to persist among vulnerable groups in Canada, including newcomer families. This mixed-methods study uses an exploratory sequential design to characterize the food security status of newcomer families with children aged 3–13 years. Parents completed food security and 24-hour dietary recall questionnaires, and parents and service providers were interviewed to explore their food insecurity experiences. Fifty percent of participant households experienced food insecurity, while 41% of children were food insecure. More recent newcomer families, and families with parents that had completed high school or some years of postsecondary training, more commonly experienced household food insecurity, compared to families with parents without high school diplomas or those with university degrees. Food-insecure children aged 4–8 years were at higher risk of consuming a lower proportion of energy from protein, lower servings of milk products, and inadequate intakes of vitamin B_12_ and calcium. Participants identified changes in food buying habits due to low income, using food budgets to purchase prescription drugs and to repay transportation loans, while the school food environment impacted children’s food security. Food security initiatives targeting newcomers may benefit from building on the strengths of newcomers, including traditional dietary practices and willingness to engage in capacity-building programming.

## 1. Introduction

Food is a human right according to the Universal Declaration of Human Rights [1], the International Covenant on Economic, Social and Cultural Rights [2], and the Convention of the Rights of the Child [3]. Canada is a signatory to all of these international agreements; however, Canada has a questionable track record with regards to upholding economic rights, such as the right to food [4]. Canada ranks 12th according to global human development indicators [5], meaning it is not among the states that prioritize economic rights.

It is not acceptable that food insecurity exists in Canada—a high-income country. However, food insecurity continues to persist, especially among vulnerable population groups, such as newcomers. The 2014 Canadian Community Health Survey (CCHS) Household Food Security Module found that 12.0% of households experienced some level of food insecurity, with Saskatchewan being the province with the lowest level of food insecurity at 10.6% [6]. Across Canada, newcomers who have been in Canada for less than five years more commonly experienced food insecurity (15.2%), as compared to newcomers who have been in Canada longer (12.0%), and the Canadian-born (11.8%) [6]. Overall, these findings indicate that recent newcomers are at increased risk for food insecurity; however, the CCHS may underestimate food insecurity among newcomers as individuals who cannot speak English or French are not included in the survey. In addition, the CCHS does not differentiate between immigrants and refugees. Other research provides more details about the depth and breadth of food insecurity among specific newcomer groups. 

In a Canadian study, Vahabi et al. [7] reported that 56% of Latin American newcomer households were food insecure, and 84% of very recent Latin American newcomer households (less than 1 year) were food insecure. Similarly, Henderson et al. [8] found that 63% of newcomers who had been living in the north end of Winnipeg for up to 6 years were food insecure. These studies used the same data collection tool as the CCHS; however, participants were surveyed in their first language, which may have supported better comprehension of the questions and resulted in more accurate responses. According to several American and Australian studies, the prevalence of food insecurity among recent newcomers ranges from 72 to 90% of households [9,10,11]. In addition, south–south migrants have been observed to be highly food insecure, including 85% of Zimbabwean immigrant households surveyed in South Africa [12]. These studies indicate that food insecurity may be common among newcomers to Canada, other high-income countries, and the global south.

Several studies have described how low income affects food-buying behaviour and subsequent consumption patterns. Food-insecure households often try to stretch the budget a little further by purchasing cheap high calorie foods [13,14,15]. Latino immigrant families have described how their diet changes with severity of food insecurity [16]. Some Latino immigrants reported that, with mild food insecurity, families try to keep the size of meals the same by relying on cheaper foods. Respondents described how they cut back on unnecessary foods (snacks and soda) and expensive foods (fruit and meat) when they run short of funds. Respondents also reported substituting less expensive foods like fruit drink powder for fruit juice. As food insecurity increases, adults eat smaller portions to allow their children to eat enough. If food insecurity becomes severe, children’s portions may be decreased and adults may not eat for a day [16]. Parents appear to try to cushion the impact of food insecurity on their children by absorbing the impact of scarce resources as long as they can.

Nutrient deficiencies and other poor health outcomes have been associated with food insecurity. A national Canadian study observed that food insecure adults and adolescents were more likely to have inadequate intakes of some nutrients [17]. Children in food insecure families were not observed to be at higher risk for inadequate nutrient intakes; however, they consumed more energy dense diets. This can have long-term health consequences as children may establish unhealthy eating patterns during long periods of household food insecurity [18]. These dietary patterns established during childhood may persist through to adulthood and contribute to excessive weight gain [19], as well as early signs of chronic disease during childhood [20]. Indeed, food insecurity has been linked with excess energy intake and subsequent weight gain among children and adults [21,22], although the results are not consistent for children [23].

In addition to nutritional concerns, Canadian research has demonstrated that food insecurity has broader public health impacts. The experience of hunger during childhood has been linked to a greater likelihood of developing certain conditions, such as asthma and depression in adolescence and early adulthood [24,25]. Adults in food-insecure households experience higher rates of chronic conditions, including heart disease, depression, and type 2 diabetes [26,27,28,29,30,31]. Among adult immigrants, food insecurity has been linked with emotional and physical health problems, including depression, anxiety, and gastrointestinal and respiratory problems [32,33,34], while food-insecure child immigrants are more vulnerable to the development of learning disabilities [35]. According to the available evidence, experiencing long-term food insecurity is linked to poor health.

In regards to food insecurity in Canada, Tarasuk et al. [6] note, “Although there has been rigorous measurement and monitoring of household food insecurity in Canada since 2005, the problem has not abated.” Simply measuring the prevalence of food insecurity has not resulted in noticeable changes. A mixed methods approach that brings together qualitative approaches and quantitative data may provide generalizable data that can more effectively support advocacy for the development of policies and programs to strengthen food security [36]. In addition, the available research regarding food security among newcomers does not differentiate between the experiences of refugees and immigrants, or subgroups of immigrants by region of origin or ethnic minority status, making it difficult to determine which group is at higher risk for food security. The authors were not able to locate an article that used a rigorous mixed-methods study design to measure food insecurity prevalence, analyze links to predictive variables, and add context with qualitative data. Accordingly, this study seeks to use a mixed-methods study design to measure the prevalence of food insecurity among Canadian newcomers, analyze possible predictive factors, including immigration status, and use qualitative data to contextualize and explain the concept of food insecurity among newcomers.

## 2. Materials and Methods

This study is part of the larger Healthy Immigrant Children Study [37] that employed a mixed-methods design using a combination of quantitative and qualitative methods in alignment with the critical realist methodological approach [38]. An explanatory sequential design was used to first yield quantitative data regarding the prevalence of food insecurity, analyze associations with predictive variables, and describe the diet of food-secure and food-insecure children, followed by qualitative data collection to produce complementary data to explain possible mechanisms underlying the quantitative data [39]. This process allowed for methodological triangulation to support a more complete description of food insecurity among newcomer families.

Quantitative data were collected through questionnaires coTncerning socio-economic status, diet, and food security. Qualitative data were gleaned through in-depth interviews with the parents of newcomer children and service providers regarding dietary changes and access to healthy food. Information gathered through the questionnaires allowed for the determination of food insecurity prevalence among newcomer families, analysis of possible associations with predictive variables, and dietary description, while the in-depth interviews contextualized and explained the experience of food insecurity. The University of Saskatchewan Research Ethics Committee provided ethical approval.

### 2.1. Participant Recruitment

The Healthy Immigrant Children study involved a cross-sectional study of 300 immigrant and refugee children aged 3 to 13 years who had been living in Regina or Saskatoon, Saskatchewan, Canada for less than five years. Study participants were purposefully selected to proportionally represent the sex, ethnicity, and countries of origin of current Saskatchewan newcomers. During the study time period, Saskatchewan received newcomers from Asia and the Pacific (68.7%), Africa and the Middle East (9.6%), Europe and the United Kingdom (12.0%), the United States (4.8%) and South and Central America (4.9%) [40]. Only healthy children, not currently being treated for malnutrition or other serious medical problems, participated in the study.

Participant recruitment was supported by several newcomer settlement organizations, such as Open Door, the largest settlement agency in Saskatchewan, who invited parents to nominate their children. Parents provided written consent, and confirmed that their children assented to participate. Interviews were conducted in English, and interpreters were available to assist as needed.

### 2.2. Quantitative Data Collection and Analysis

Income-related food security was evaluated using the Household Food Security Survey Module (HFSSM), which Statistics Canada has used since 2004 in Canadian Community Health Surveys (CCHS). The HFSSM uses participant self-reporting by adults in response to questions on insufficient food access, availability and utilization related to family financial limitations over the previous 12 months. The 18 questions range from asking about concern with running out of food to changed eating patterns as a result of financial limitations. Ten of the 18 questions correspond to adult experiences of food security or household food security, while eight correspond to children’s experiences of food security in the household [41]. The food security module has been validated in 19 countries including Canada [42]. Participant households were categorized as food secure, marginally food insecure, moderately food insecure, and severely food insecure.

To determine usual intake, each child completed three 24-hour dietary recalls spaced at least 10 days apart [43]. Accurate descriptions and portion sizes of foods consumed were facilitated through use of food pictures and diagrams. Parents were asked to provide ingredients for mixed dishes. Each participant’s usual intake was obtained through averaging the three 24-hr dietary intakes. All 24-hr recalls were administered in person due to language barriers and difficulties with describing portion sizes over the telephone. This methodology is similar to the pilot study with participants of the same age range, as well as another study by Vatanparast [44].

The diet analysis program Food Processor Nutrition and Fitness Software version SQL 10 (Esha Research, Salem, OR, USA) was used to determine intake of food group portions and specific nutrients. Although more than 5000 Canadian food items are included in this program, if a specific food was not included, the American food item most similar in nutrient content was substituted. The results were analyzed to compare the number of servings of vegetables and fruit, grain products, milk and alternatives, and meat and alternatives consumed by food-secure and food-insecure children. Prevalence of micronutrient inadequacies was calculated using the percent below EAR approach based on the Dietary Reference Intakes for age and sex categories for food-secure and food-insecure children. 

Descriptive data are presented as means ± standard deviations to compare refugee children to immigrant children. Data were tested for normal distribution using the Shapiro–Wilk test and, when found to be not normally distributed, data were either transformed or subjected to equivalent non-parametric tests (e.g., Mann–Whitney U-test). A 2-sided independent Student’s t-test was used to evaluate differences between immigrants and refugees, and food-secure and food-insecure children. A multivariate logistic regression model was used to investigate associations between food security and possible predictive variables. Statistical best fit model building included univariate analysis to determine a subset of explanatory variables, subsequent multivariate analysis with the elimination of variables that did not contribute to the best fit, exploring interactions between variables, and checking for confounding variables. Statistical analysis was conducted using the Statistical Package for the Social Sciences (SPSS 25, IBM, Markham, ON, Canada). Alpha was set at 0.05 for all tests. 

### 2.3. Qualitative Data Collection and Analysis

The second phase of the study involved inviting a purposefully selected sample of the participants’ parents to participate in in-depth interviews to better understand newcomers’ access to healthy foods. A diverse selection of 19 refugee and immigrant families of various ethnic and socio-economic backgrounds participated in in-depth individual or household interviews. Household interviews included any combination of consenting household members, including parents, their children and extended family members. The semi-structured in-depth interviews focused on dietary changes and barriers to healthy eating. The parent interview guides included questions regarding their children’s general health, factors affecting their health, their children’s diet, and probes for barriers to eating a healthy diet. Interviews used open-ended questions to facilitate the collection of rich, descriptive narratives [45] until further interviews did not yield significant information beyond that already collected, indicating saturation had been reached. 

In addition, a purposefully selected sample of newcomer service providers, healthcare providers, and policy makers was invited to participate in in-depth interviews to understand their perspectives on newcomer food security. Twenty-five participants representing organizations that interact with newcomers in a variety of settings and at different levels were invited to participate. They included 23 service providers from settlement agencies, community schools, English-as-a-Second-Language programs, food banks and healthcare organizations, as well as two provincial government policy and program consultants. Similar to the in-depth interviews with newcomers, interviews were conducted to the point of saturation. The parent interview guide questions were rephrased for the service provider interview guide to reflect that the questions referred to their experiences with newcomer children in general, not their own children, as well as asking about programming related to food and nutrition. 

Interviews were recorded and transcribed verbatim following the sessions. Transcripts were then rechecked against the audiotapes at minimum a second time by the same person. In-depth interview data were analyzed using an inductive approach with open coding of early data to generate categories embedded in the data in alignment with grounded theory [46]. Data analysis was conducted concurrently with early interviews in an iterative process so initial results could be used to fine tune participant questions and probing. As recommended by Morse [47], the context of the knowledge gained from all previous interviews influenced interpretive coding decisions by the primary researcher. Deviant cases were thoroughly reviewed to draw out the diverse experiences of participants. 

Coding categories were generated from salient data extracts corresponding to the research question. Code categories were reviewed to identify main themes and code categories were combined under the main themes. Themes were identified by referring to both the frequency of similarly coded data extracts, as well as divergent experiences to explore the range of experiences. This process facilitated the transformation of individual narratives into a critique of social processes and structures that organize lived experiences. Qualitative data analysis was done using NVivo11.

Rigor was achieved through continuing interviews to the point of data saturation, probing for expansion of ideas and prolonged engagement to ensure thick description [47]. In addition, triangulation of data from two different sources, parents and service providers, increased validity by theoretically increasing the sample size as service providers were drawing on their experience with a large number of newcomers. An inductive approach with attention to both positive and negative experiences was used to minimize researcher bias.

Purposeful sampling to obtain a sample representative of the ethnic origins of recent Saskatchewan newcomers contributed to data reliability in the Saskatchewan context. The attainment of internal reliability was evident by the overlapping interview data from different participants. 

As per the explanatory sequential design, quantitative data were analyzed, followed by qualitative data to produce complementary data [39]. The qualitative and quantitative data were then merged through a side by side comparison to explore areas of convergence or triangulation and to contextualize and explain the quantitative results [38,48]. The in-depth interview data provided rich description of lived individual experiences, and protected against the possibility of proceeding down a reductionist path with unwarranted categories and priorities suggested by quantitative data [49]. Merging the quantitative and qualitative data supported the development of a robust data set that described prevalence and predictive variables along with the contextualization of lived experiences.

## 3. Results

### 3.1. Quantitative Results

Participant demographics are available in Table 1. Participants commonly originated from Asia (49%), the Middle East (28%), Africa (12%), Latin America (4%), Eastern Europe (4%) and Western Europe or United States (3%). Although 79% of all participants were in the low- or middle-income categories, a higher proportion of refugees (92%) were in these categories compared to immigrants (65%). In addition, refugees more commonly had lower levels of education. 

Food security status was determined at both the household and child level. As per Table 2, 50% of participant households experienced food insecurity, with 18% being marginally food insecure, 26% being moderately food insecure, and 6% being severely food insecure. Forty-one percent of children were food insecure with 16% being marginally food insecure, 24% being moderately food insecure, and 2% being severely food insecure.

Significant predictors of household food security included length of residence in Canada and parents’ education level (Table 3). Recently arrived newcomer families and families that included parents that had completed high school or some years of postsecondary training were at high risk for household food insecurity compared to families that included parents that had not completed high school or had a university degree. Similarly, at the level of child food insecurity, children who were part of families who have more recently arrived or had parents that had completed high school or some years of postsecondary training were at high risk for food insecurity.

Children’s mean energy density and macronutrient composition did not significantly differ in relation to household food security status among most age groups (Table 4). However, food-insecure children aged 4–8 years consumed a lower proportion of energy from protein, which was replaced with energy from carbohydrate, compared with the food-secure children. Children’s consumption of servings from the different food groups did not significantly differ in relation to household food security status among all age groups, except for milk products consumption among the 4–8-year-old group (Table 5). However, in more than half of the measurements, food-insecure children consumed a lower number of average servings than food-secure children. In regards to prevalence of inadequacy for select nutrients, food-insecure children experienced a higher level of inadequate nutrient intake, such as vitamin D and calcium, than food-secure children in 75% of the measurements (Table 6). Significantly more food-insecure children in the 4–8-year-old group consumed inadequate amounts of vitamin B_12_ and calcium, while significantly more food-secure females in the 9–13-year-old group consumed an inadequate amount of iron. 

### 3.2. Qualitative Results

All participants were either parents of children between the ages of 3 and 13 years or newcomer service providers. Interviewees included parents from 19 distinct family units, made up of 15 mothers and 7 fathers, and 13 immigrants and 9 refugees. Both the mother and father participated as a couple in 3 cases. Participants were from the Middle East (8), Asia (6), Africa (2), Latin America (1), Eastern Europe (1), Western Europe (2), and the United States (2). The vast majority reported low incomes. In contrast, families from the United States and Western Europe reported high incomes, while one Middle Eastern and one Latin American family reported middle incomes. Service providers were not asked to provide demographic information. 

Although many newcomers were reluctant to comment on not having sufficient access to food, a few refugees mentioned changes in food buying habits related to decreases in income and trying to stretch food a little further. A Saskatoon refugee from Asia (R1) shared, “*…they (children) do drink milk and when we used to get assistance from the government we used to buy big gallons, now we are on our own so we buy in 1 liter cartons.”* Similarly, another Saskatoon refugee from Asia (R2) commented, “*The family shops mostly weekly…the children eat whenever they want…and dad cannot provide the amount they want to eat. It is expensive and dad’s budget is $600 to $700 per month so dad tells kids it is OK to eat, but make it available for the next day also, so eat in a controlled way.*” A Saskatoon refugee from the Middle East (R3) clarified, “*Before they gave us a few hundred dollars for food so it was pretty good…we bought good things…the government gave us an allowance when we first came, but then after it is done some people have problems with having enough money for food. When we came they gave us a good amount to buy what we needed for the house, furniture. Only the first 2 months felt like we had enough money and then after that it was difficult. After that we only had a small monthly amount.*” However, none of the immigrant families mentioned any difficulties with food security. A Regina healthcare provider (HC1) added further perspective, “*…some families can’t afford to have red meat. They may afford to buy pasta, white bread and potatoes, things that are cheap to cook for them.*”

For some newcomers, food security was balanced by the need to make prescription drug and hygiene product purchases not covered by benefit programs. The son of a Saskatoon refugee from the Middle East (R3) shared, “*When the ear pain was really bad we had to buy the medicine after 2 days when the child tax credit came. My mom never ever uses our money except for food, but it was really important, she had to because it hurt a lot.”* A Saskatoon immigrant service provider (SP1) further explained, “*… $255 (adult) living allowance…a female she needs feminine stuff, she cannot buy anything extra…even if she needs to buy Tylenol, Advil, $9, $10, $11 from this, she wants to buy lotion, shampoo, everything comes from this $255. So they have to use at least one of the child’s tax benefit to buy those things, so they want to feed the children to fill their stomach, not vitamin C and D…they cannot.*”

In some cases, insufficient prescription drug benefits impacted food security over the long-term for those with chronic health conditions. An immigrant service provider (SP1) described her client’s situation:

I have a client, mother and son with HIV. HIV medication is very expensive and she has to pay 2% and the 2% for her and her son she was paying $149 every month…so she is paying from her food allowance $74 and her son is paying from the child tax benefit $74 every month. The price of the drug is close to $1,400, very expensive medication…the doctor is always telling her healthy food and activity. She says if I pay $75 every month from my food budget, where I will get the money to eat healthy food? She is eating lentils and enjera…Also chickpeas, all vegetarian, cabbage, potato, because there is not enough money…She gets $40 extra for food, and only if the doctor writes a note for good nutrition…From $1,400 she is paying not even 2%, its 1%, but 1% according to the scale of amount is a lot for her. Why they don’t forgive 1%?

Although government-sponsored refugees can generally depend on some support through social assistance and drug benefit programs immediately upon arrival, immigrants do not have the same access to all these supports. When they arrive, they are expected to have enough money in their bank account to support themselves for at least 6 months until they can find work, so they are not eligible for social assistance and they are often not aware of health benefit programs when they first arrive. A Saskatoon immigrant service provider (SP2) commented, “*With immigrants…if they are coming through the economic class they are expected to have about $12,000 to $15,000…in their bank account. This is about enough for surviving about 6 months, but the reality is there is always the fear of running out of money…a lot of parents…who sacrifice…eating healthy, because…they have a very limited bank account and they are scared of running out of money.*”

In addition to prescription medicine, refugees encountered financial pressures related to repayment of transportation loans provided by the Government of Canada that impacted their food budget. A Saskatoon immigrant service provider (SP1) stated, “*…they have to pay back their transportation loan…a family of 4…it costs them close to $7000…and they have to pay that out of their food, the $255 living allowance. The government starts taking that back right away, 3 or 4 months after they arrive.*”

In addition to income limitations, children’s food security can be affected by the school food environment if children feel uncomfortable eating their lunch at school. The school environment can have a profound effect on a newcomer child’s daily dietary intake. A Saskatoon immigrant service provider (SP3) noted:

There was a family…the mother is always giving him (son) sandwich…and after a year…under the stairs of the apartment it was smelling bad …and then they found it under the stairs, the food was there for the whole year…he told them ‘I don’t want to eat lunch because the sandwich is not good. When I taste it from other kids it tastes so good…I don’t like your sandwich.’

When contacted to ask about food security initiatives for newcomers, the Regina Food Bank advised that 6% of their clients who collect food hampers self-identified as newcomers to the community. They described how they make efforts to lower barriers for newcomers to access their services by accepting confirmation of permanent residency documents in place of the normally required Saskatchewan Health Card as proof of residency, as well as providing ongoing services for a one year period following initial intake before having to meet with a client intake worker again. In collaboration with other immigrant serving organizations, the Regina Food Bank stated that they have branched out to providing training of interest to newcomers through their Adult Centre for Employment Readiness and Training (ACERT). They have offered English as a Second Language, Workplace Essential Skills for Newcomers, computer training, job search workshops and Nutritional Leadership Cooking training programs. The Nutritional Leadership Cooking program provides basic instruction on healthy, affordable food selection and preparation. In 2013, about 60% of ACERT clients self-identified as an immigrant or refugee. In addition, the Regina Food Bank is part of a Newcomer Food Security Group with other community partners. This group meets about four times a year to discuss newcomer food security issues and to plan activities that support newcomers.

## 4. Discussion

The current study captures issues of food insecurity that extend beyond low income, including insufficient access to health benefit programs, and the requirement for government assisted refugees to initiate prompt repayment of transportation loans. Further, participant recollections about newcomer children who do not eat or who throw away the lunch they bring to school because they are embarrassed about their ethnic food, provides an additional dimension to the concept of food security for children related to the school food environment, where “junk” foods (e.g., pop, cookies, chips, candies) may be widely available [50]. 

Consistent with other Canadian studies [7,8], 50% of participant households experienced some level of food insecurity and food insecurity was more common among more recent newcomer families. However, the prevalence of food insecurity observed in the present study and the other two Canadian studies [7,8] are much higher than the prevalence recorded through the CCHS survey (15.2%), which only includes individuals who can speak English or French, thus not reaching potentially vulnerable individuals. Enhanced outreach by community partners to recruit newcomers, along with use of interpreters to support the participation of individuals who did not speak English or French may have inadvertently resulted in increased participation by vulnerable newcomers who are usually difficult to recruit by telephone. In addition, the high level of trust built with study participants may have led to more accurate responses in the current study. Many participants were initially reluctant to complain because they were grateful for the opportunity to be in Canada and perhaps fearful of consequences associated with admitting to their struggles. 

Low income did not emerge as a predictive factor of food insecurity in the current study, possibly because of homogeneity of the sample where a large majority of the participants were low income so there was not enough variation to demonstrate an association. However, low income has been linked to food insecurity in Canada and other high-income countries [6,51]. Refugee parents’ comments related to income appear to indicate that they felt well supported when they first arrived and received a fair sum of money to purchase all of their initial household needs. After this initial period, they received a smaller monthly amount to cover food and other monthly expenses, which some families did not perceive as adequate. Then, once they started working at low wage jobs, some families found they had less money for food and tried to stretch their food dollars a little further. These findings appear to indicate that the association between food security and length of stay among refugees may be more of a U-shape with food security being initially high, then decreasing as the family income sources change and perhaps become less stable, and finally food security increases as the family’s financial situation improves and stabilizes.

The finding that families with parents that have completed high school or some years of postsecondary training were at higher risk for household food insecurity may be related to difficulties with navigating available community resources and social safety net benefit programs because they are not accustomed to living in resource-poor situations. Families with less than high school-level education may be more adept at managing with lower incomes. This finding may also be confounded by collective social functioning and social capital, which was not measured in the current study. High social collective functioning and social capital have been linked to increased capacity to access food and being food secure [51,52]. Families with less than high school education may be quicker to rebuild their social support network in Canada out of necessity. Families with parents that have university degrees may be more food secure because some of them immigrated to Canada in response to job offers so they have stable incomes. Studies among the general population often find that less education is associated with food insecurity [51]. However, some newcomer families with low education levels have lived on low incomes for extended periods and have developed coping skills that often include accessing social networks. 

According to comments related to other expenditures cutting into the food budget, food security is not just about having enough money to purchase healthy food. For refugees, access to adequate prescription drug benefits and having some control over the timing and amount of transportation loan repayments could have a substantial impact on food security. Immigrants could benefit from being informed about benefit programs like the rental housing supplement and family health benefits. Much of the available research on food security does not provide details about how other financial pressures may be impacting food security.

All of the participant comments related to food security came directly from or were concerning individuals from ethnic minorities. None of the participants from the United States or Western Europe mentioned any concerns about having sufficient funds to purchase healthy food. This finding is consistent with Patricia Hill Collins’ web of oppression [53] that describes an interlocking structure of domination that encompasses all identity variables. Hill Collins describes how variables of race, class, gender, age, sexual orientation, religion and ethnicity form an interlocking system that bestows varying amounts of privilege and oppression on individuals. Ability to speak the official language could also be added as an identity variable that impacts privilege. Most refugees and many immigrants from ethnic minorities arrive in Canada with very limited resources and have a long struggle ahead of them as they seek to overcome the many obstacles to establishing safe and comfortable lives for their families.

Food insecurity during childhood can have a variety of negative impacts that may persist into adulthood. In addition to the international research indicating that young children in chronically food-insecure households may experience adverse health outcomes in childhood or adulthood [19,54,55], recent Canadian research has shown that children who experience food insecurity are at risk for physical and mental health problems, such as depression and asthma in adolescence and early adulthood [24,25]. The high prevalence of food insecurity among newcomer families in the current study raises concerns that newcomer children are at high risk of experiencing adverse health impacts over their life course.

There were not many significant findings concerning children’s mean energy density, macronutrient composition and dietary inadequacies in relation to household food security status; however, some measurements indicate areas for future research. Overall, food-insecure children aged 4–8 years appear to be more vulnerable to poor diet quality, as indicated by significantly lower proportion of energy from protein, lower servings of milk products, and higher prevalence of inadequate intakes of vitamin B_12_ and calcium compared to food-secure children. The high prevalence of some nutrient inadequacies, such as folate, vitamin D, calcium and zinc, among both food-secure and food-insecure children suggests that observed nutrient inadequacies may be more related to dietary patterns among newcomer families, rather than food security. The finding that significantly more food-secure females aged 9–13 years consumed an inadequate amount of iron may be related to emerging body image concerns that impact dietary patterns and/or increased consumption of fast foods with poor iron content among food-secure adolescent females [56].

While no government ministry or organization claims responsibility for assuring the food security of Saskatchewan’s population, there is a patchwork of organizations that provide food hampers, school lunches and ready-made meals. The fact that only 6% of the traditional Regina food bank clients were newcomers may indicate that newcomers are not aware of or do not know how to access food bank services. However, the additional finding that 60% of ACERT clients at the Regina food bank were newcomers, may indicate that they are more receptive to capacity-building programming as compared to charitable food donations. Organizations seeking to improve food security among newcomers should likely focus on skill-building programming to increase employment options and healthy food preparation skills, as well as orientation to existing options, such as food banks that can assist with emergency food needs.

Although this study did not measure level of acculturation—the degree to which traditional culture is supplanted by host country cultural practices—it is important to acknowledge that level of acculturation may mediate the impact of low-income on the development of food insecurity among newcomer children [57]. There is also a cultural dimension to food security that includes limited access to preferred traditional foods; shopping difficulties in a new food environment; and lack of knowledge regarding how to use unfamiliar foods, such as canned foods [58]. Initiatives aiming to improve newcomers’ food security status should incorporate the important role of maintaining traditional cultural dietary practices and learning new cooking skills to positively impact food security among low-income families. Future studies regarding food security should include measures of acculturation and dietary patterns to understand the role of traditional dietary customs.

## 5. Conclusions

Food insecurity continues to be a concern for newcomers, especially refugee families. Newcomer families vulnerable to food insecurity are often headed by parents that have completed high school or some years of postsecondary training and are in the early stages of integration into the Canadian work environment. Food-insecure children aged 4–8 years appear to be at higher risk for dietary inadequacies. Beyond low income, food insecurity is linked to lack of awareness of health benefit programs, insufficient drug benefits, insufficient funds for hygiene items, and repayment of government transportation loans provided to refugees. The results suggest policy options that could improve food security among newcomers. 

## Figures and Tables

**Table 1:** Participant demographics.

Characteristics	Immigrants *n* = 134 (44.7%)	Refugees *n* = 166 (55.3%)	All Participants *n* = 300 (100%)
Age (Mean ± Standard Deviation [SD])	8.3 ± 2.9 *	7.8 ± 2.7	8.0 ± 2.8
**Sex**			
Male	75 (56.8%)	102 (61.4%)	177 (59.4%)
Female	57 (43.2%)	64 (38.6%)	121 (40.6%)
**Region of origin**			
Middle East (E.g. Iran, Iraq, Pakistan)	65 (49.2%) *	19 (11.4%)	84 (28.2%)
Asia (E.g. Burma, India, Philippines)	33 (25.0%) *	114 (68.7%)	147 (49.3%)
Africa	13 (9.8%) *	22 (13.3%)	35 (11.7%)
Latin-America	2 (1.5%) *	11 (6.6%)	13 (4.4%)
Eastern Europe	11 (8.3%) *	0	11 (3.7%)
Western Europe/US	8 (6.1%) *	0	8 (2.7%)
**Length of stay in Canada in years (Mean ± SD)**	2.0 ± 1.6 *	2.6 ± 1.5	2.3 ± 1.6
**Education**			
Neither parent has high school diploma	7 (5.3%) *	128 (80.0%)	135 (46.2%)
At least one parent has high school diploma, some university or other education	27 (20.5%) *	19 (11.9%)	46 (15.8%)
At least one parent has university degree	98(74.2%) *	13 (8.1%)	111 (38%)
**Main source of income**			
Wages and salaries	114 (86.4%) *	88 (53.7%)	202 (68.2%)
Social assistance and Gov’t Transfer	4 (3.0%) *	75 (45.7%)	79 (26.7%)
Other (Scholarship, savings, none)	14 (10.6%) *	1 (0.6%)	15 (5.1%)
**Income Category (Adjusted for number of family members as per Canadian Community Health Survey CCHS)**			
Lowest	37 (30.3%) *	58 (41.4%)	95 (36.3%)
Middle	42 (34.4%) *	71 (50.7%)	113 (43.1%)
Upper-middle	29 (23.8%) *	10 (7.1%)	39 (14.9%)
Highest	14 (11.5%) *	1 (0.7%)	15 (5.7%)
**Low Income Cut Off (LICO)**			
Income below LICO (Using # persons per	66 (54.5%) *	108 (77.1%)	174 (66.7%)
Income above LICO household)	55 (45.5%) *	32 (22.9%)	87 (33.3%)

* indicates significant difference between immigrants and refugees through x^2^ at *p* < 0.05.

**Table 2:** Food security status of participants.

**Characteristics**	**Immigrants** ***n* = 127 (44.7%)**	**Refugees** ***n* = 157 (55.3%)**	**All Participants** ***n* = 284 (100%)**
**Household Food Security**			
Food Secure	72 (56.7%) *	70 (44.6%)	142 (50.0%)
Marginally Food Insecure	26 (20.5%) *	26 (16.6%)	52 (18.3%)
Moderately Food Insecure	25 (19.7%) *	49 (31.2%)	74 (26.1%)
Severely Food Insecure	4 (3.1%) *	12 (7.6%)	16 (5.6%)
	**Immigrants** ***n* = 127 (44.7%)**	**Refugees** ***n* = 157 (55.3%)**	**All Participants** ***n* = 284 (100%)**
**Child Food Security**			
Food Secure	88 (69.3%) *	79 (50.3%)	167 (58.8%)
Marginally Food Insecure	16 (12.6%) *	29 (18.5%)	45 (15.8%)
Moderately Food Insecure	21 (16.5%) *	46 (29.3%)	67 (23.6%)
Severely Food Insecure	2 (1.6%) *	3 (1.9%)	5 (1.8%)

* indicates significant difference between immigrants and refugees through *x*^2^ at *p* < 0.05.

**Table 3:** Predictors of food insecurity *.

Variable	Odds Ratio	95% Confidence Interval	*p*-Value
**Household food insecurity**			
Length of residence	0.706	0.578–0.863	0.001
**Parents’ education level**			
Base: < high school diploma			
High school diploma or some university	3.324	1.231–8.977	0.018
University degree	0.865	0.344–2.324	0.758
Constant	0.777		
Food secure (0) vs. food insecure (1) best fit logistic regression model. Other confounders that remained in the final logistic regression model include the number of children in the household, region of origin, immigration status and income category.			
**Child food insecurity**			
Length of residence	0.729	0.591–0.899	0.003
**Parents’ education level**			
Base: < high school diploma			
High school diploma or some university	2.930	1.078–7.965	0.035
University degree	1.629	0.592–4.484	0.345
Constant	1.350		
Food secure (0) vs. food insecure (1) best fit logistic regression model. Other confounders that remained in the final model for child food insecurity include number of children in the family, region of origin, immigration status and income category.			

* Food-insecure category includes combined marginal, moderate and severe food insecurity.

**Table 4:** Children’s mean energy density and macronutrient composition from 24-hr recall average in relation to household food security status *.

	3 Years (*n* = 17)		4–8 Years (*n* = 137)		Males 9–13 Years (*n* = 63)		Females 9–13 Years (*n* = 54)	
	Intake	*p* Value	Intake	*p* Value	Intake	*p* Value	Intake	*p* Value
Energy intake (kJ)								
Food secure	1404 ± 226 ^1^	0.05	1538 ± 396	0.36	2007 ± 625	0.06	1489 ± 442	0.33
Food insecure	1025 ± 356		1471 ± 458		1710 ± 579	1489 ± 442	1616 ± 505	
Energy density (kJ/g)								
Food secure	4.7 ± 0.1	0.76	4.8 ± 0.2	0.26	4.8 ± 0.2	0.83	4.7 ± 0.2	0.94
Food insecure	4.7 ± 0.4		4.7 ± 0.2		4.8 ± 0.2		4.7 ± 0.2	
Proportion of energy from protein (%)								
Food secure	13.4 ± 3.2	0.72	15.2 ± 3.6	0.03	14.2 ± 3.0	0.26	14.3 ± 3.0	1.0
Food insecure	12.7 ± 3.8		14.0 ± 2.8		15.0 ± 2.8		14.3 ± 2.1	
Proportion of energy from carbohydrates (%)								
Food secure	59.6 ± 3.8	0.88	55.4 ± 8.1	0.08	54.5 ± 7.9	0.78	58.7 ± 8.2	0.74
Food insecure	58.9 ± 10.8		57.8 ± 7.3		54.0 ± 7.3		58.0 ± 6.1	
Proportion of energy from fat (%)								
Food secure	28.8 ± 2.6	0.67	30.6 ± 6.6	0.31	32.3 ± 6.0	0.93	28.0 ± 7.3	0.51
Food insecure	31.0 ± 10.7		29.5 ± 5.8		32.1 ± 6.6		29.1 ± 5.6	

^1^ Values are means ± SD using independent samples t-test. * Food-insecure category includes combined marginal, moderate and severe food insecurity.

**Table 5:** Children’s consumption of foods from Canada’s Food Guide to Healthy Eating food groups from 24-h recall average in relation to household food security status *.

	3 Years (*n* = 17)		4–8 Years (*n* = 137)		Males 9–13 Years (*n* = 62)		Females 9–13 Years (*n* = 54)	
	Servings	*p* Value	Servings	*p* Value	Servings	*p* Value	Servings	*p* Value
Fruits and Vegetables								
Food secure	4.5 ± 3.6 ^1^	0.67	4.0 ± 2.0	0.35	3.9 ± 2.4	0.72	4.4 ± 2.3	0.91
Food insecure*	3.9 ± 1.9		3.7 ± 1.7		3.7 ± 1.8		4.4 ± 1.6	
Milk and Alternatives								
Food secure	1.8 ± 0.8	0.48	1.7 ± 1.2	0.04	1.8 ± 1.3	0.21	1.2 ± 1.0	0.54
Food insecure	1.3 ± 1.3		1.3 ± 0.9		1.4 ± 1.0		1.3 ± 0.8	
Meats and Alternatives								
Food secure	1.0 ± 0.6	0.88	1.6 ± 0.8	0.28	2.2 ± 1.3	0.41	1.4 ± 0.8	0.51
Food insecure	1.0 ± 0.8		1.4 ± 0.9		2.0 ± 0.8		1.6 ± 1.0	
Grain Products								
Food secure	3.9 ± 1.4	0.06	4.9 ± 2.1	0.85	6.2 ± 2.4	0.76	5.2 ± 2.0	0.73
Food insecure	2.5 ± 1.3		5.0 ± 1.8		6.0 ± 2.8		5.3 ± 1.8	

^1^ Values are means ± SD using independent samples t-test. * Food-insecure category includes combined marginal, moderate and severe food insecurity.

**Table 6:** Prevalence of inadequacy among children for select nutrients ^.

	Protein	Folate	Vitamin B_12_	Vitamin D	Calcium	Iron	Zinc
	Prevalence of Inadequacy (%) ^1^						
3 years (*n* = 17)							
Food secure	0	0	0	100	20.0	0	0
Food insecure	0	33.3	8.3	75.0	66.7	8.3	25.0
4–8 years (*n* = 137)							
Food secure	0	15.9	0	88.4	66.7	4.3	7.2
Food insecure	0	22.1	13.2 *	92.5	82.4 *	7.4	10.3
Males 9–13 years (*n* = 63)							
Food secure	5.7	40.0	5.7	82.9	82.9	8.6	34.3
Food insecure	7.4	39.3	10.7	96.4	85.7	21.4	42.9
Females 9–13 years (*n* = 54)							
Food secure	7.1	53.6	25.0	96.4	92.9	28.6	57.1
Food insecure	11.5	42.3	15.4	92.3	92.3	7.7 *	61.5

^1^ Prevalence of inadequacy was calculated using the EAR cut-point approach, with analyses stratified by age/sex group and household food security status. ^ Food-insecure category includes combined marginal, moderate and severe food insecurity. * indicates significant difference between food-secure and food-insecure children through *x*^2^ at *p* < 0.05.
